# Supplementary material for: Learning curve and surgical outcome of robotic assisted colorectal surgery with ERAS program
Source: Sci Rep. 2022 Nov 29;12:20566. doi: 10.1038/s41598-022-24665-w (PMC9709162; doi:10.1038/s41598-022-24665-w)
Supplement: Supplementary file 1 — Supplementary Information 1. [file 41598_2022_24665_MOESM1_ESM.zip › LSC Raw data-Ñ[▒K/139/H_ñw╝╨░O▒Kñσ.pdf]

正式摘要(電子簽章：0000002263981111)	☐【讀取】憑證	
W83 / W83 ☐英文標題(注意:無英文姓名-請補病人基本檔!)		

臺中榮民總醫院
出院病歷摘要

轉入號 : 轉出號 :

[redacted]

**(9)**入院日期	110/09/30 (2021) 直腸外科 W83 - 113
**(10)**轉科日期	
**(11)**出院日期	110/10/04 (2021) 住院天數計 4 日

診斷

- 入院:Cecal polyp, s/p polypectomy, proved adenocarcinoma
- 出院:Cecal adenocarcinoma, s/p laparoscopic right hemicolectomy on 2021/10/01

主訴

- Adenocarcinoma was founded in regular colonscopy health examination.

病史

- This is a 43 years old male with no underline disease. Family history was familial adenomatous polyposis in her mother and older sister.
  The patient accepted regular colonscopy every year and found cecal polypectomy*2 proved to be adenocarcinoma, MD s/p excision on 2021/09/10. in 中山H. Acoording to the patient, regular colonscopy found stage 0 adenoma in cecum s/p excisionin 2019. Besides, the patient complained the stool was hard in the recent 2 years. This time, she was admitted for Laparoscopic right colectomy and anastomosis on 2021/10/1.

體檢發現

-
  - General appearance :
    - easy looking , conscious clear , without cardiopulmonary distress
    - body weight : 52.6 kg , height : 163.2 cm
    - Vital signs自動帶入date 體溫(BT) 脈搏(P) 呼吸(R) 血壓(BP) 身高(BH) 體重(BW) BMI
      20210930 36.2℃ 82次/分 18次/分 110/74mmHg 163.2cm 52.6kg 19.7
    - Vital signs: B.P. 110/74 mmHg , P.R. 82 /min , R.R. 18 /min , B.T. 36.2 ℃
  - HEENT :
    - Eyes: no anemic conjunctiva, no icteric sclera, normal EOM
    - ENT: no otorrhea, no rhinorrhea, no erythematous mucosa, no injected tonsil, no deformity
  - Neck: supple, freely movable, trachea midline,no deformity, no engorged jugular vein , no palpable thyroid or lymph node enlargement
  - Chest:
    - Inspection: Grossly normal, symmetric and fully expansion, no accessory muscle used, no operation scar, no spider angiomata, no gynecomastia
    - Palpation: no palpable mass or nodule, no subcutaneous emphysema, no tenderness
    - Ausculation: clear breathing sound
  - Heart:
    - Palpation: no thrill, no heave
    - Percussion: normal heart size
    - Ausculation: regular heart beat, normal S1, S2, no gallop, no murmur
  - Extremities: No deformity, full ranged of motion without limitation, no clubbing finger or nail cyanosis, no pitting edema, no ulcer or pigmentation, no obvious skin lesion, no varicose vein, no bone, joint or muscle tenderness

CRS CONDITION

-
  - Karnofsky performance status: (100) Normal, no complaints, no evidence of disease
  - PE of Abdomen:
  - CEA: 1.84 ng/ml
  - Imaging Studies:
  - Colonoscope report: .
  - Figures:
    1. colon & rectum

手術日期及方法

- 手術日期及方法20211001 Laparoscopic right colectomy and anastomosis

住院治療經過

- After operation, operation with laparoscopic right hemicolectomy was done on 10/1. Under ERAS protocol, we let her try water 6 hours after operation and then clear liquid diet. IVF was declined on POD2. Under the stable condition, she was discharged on 10/4.

合併症

- nil

一般檢查

- 累積報告：

DATE	TIME	ALB	TP	BIL.T	BIL.D	ALKP	AST	ALT	LDH	AMY	LIPASE	NH3	Ferritin	UA
1101001	1309	3.9		0.7	0.2		18	11						

DATE	TIME	WBC	RBC	HGB	HCT	MCV	PLT	BLST	NEUT	BAND	SEG	LYM	MON	EOS	BAS
1101001	1311	5500		10.9			216		89.4			9.3	0.9	0.0	0.4

DATE	TIME	NA	K	CL	BUN	CREAT	eGFR	CA	Mg	P	CRP	ESR	PCT	D-Dimer	LACTATE
1101001	1309	138	4.1		9	0.62									

特殊檢查

病理報告

其它

出院時情況

- 3.改門診治療

出院指示

-

藥名	劑量	劑量單位	用法	頻次	數量	數量單位	現況	天數	單位
Allegra tab 60mg	1	TAB	PO	QN	8	TAB	TH	8	天
Mag. Oxide tab	2	TAB	PO	TID	48	TAB	TH	8	天
(口)Takepron OD tab 30mg	1	TAB	PO	QDAC	8	TAB	TH	8	天
SinphadoL tab 25mg	1	TAB	PO	TID	24	TAB	TH	8	天
Ultracet tab	0.5	TAB	PO	Q6H	16	TAB	TH	8	天

出院計劃

主治醫師_2062F 蔣鋒帆_ / 住院醫師_2331F 陳凱祥_

正式摘要(**電子簽章：000000226398111)**

W83 / W83

doctorRank:B
